# Successful treatment of erythrodermic pemphigus foliaceus with intravenous immunoglobulin^☆^

**DOI:** 10.1016/j.abd.2022.05.013

**Published:** 2023-09-22

**Authors:** Hiram Larangeira de Almeida, Junior Wieczorek, Mahony Santana, Celina Leite

**Affiliations:** a Postgraduate Program in Health and Behavior, Universidade Católica de Pelotas, Pelotas, RS, Brazil; b Department of Dermatology, Universidade Federal de Pelotas, Pelotas, RS, Brazil; c Faculty of Medicine, Universidade Católica de Pelotas, Pelotas, RS, Brazil

*Dear Editor,*

Pemphigus foliaceus (PF) is characterized by the presence of superficial vesicles or bullae in the absence of mucosal involvement, it results from the interaction of IgG autoantibodies with desmoglein 1, present in the upper layers of the epidermis.^1^ PF is endemically present in Brazil and other South American countries^1^ and can manifest as localized and disseminated forms. The disseminated forms are subdivided into four clinical variants, vesico-bullous, keratotic, herpetiform and erythrodermic. In the latter, the entire tegument is erythematous and desquamative, with areas of erosion, exudation, and crusts.^2^

This case report describes a 68-year-old female patient, who started showing erythematous-desquamative lesions on the face, upper trunk and arms, with circinate edges and lamellar desquamation (Fig. 1A). The edge of one lesion was biopsied and an upper intraepidermal cleavage was demonstrated on histopathology, along with acantholytic cells (Fig. 2), confirming the diagnosis of classic PF, since the patient does not come from an endemic area. Additional confirmation of the diagnosis was obtained through immunofluorescence, which showed an intercellular epidermal pattern of IgG deposition.

**Figure 1:**
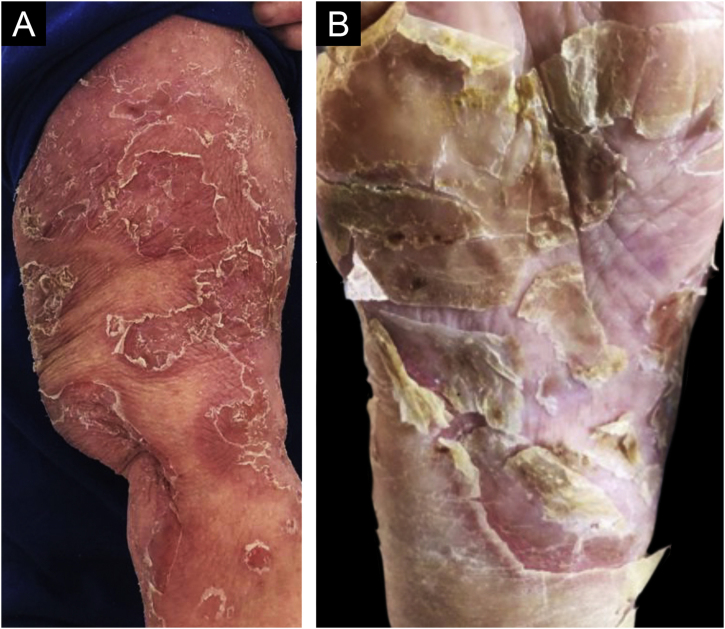
(A) Erythematous-desquamative lesions on the arm, with circinate edges and lamellar desquamation. (B) Plantar lamellar desquamation

**Figure 2:**
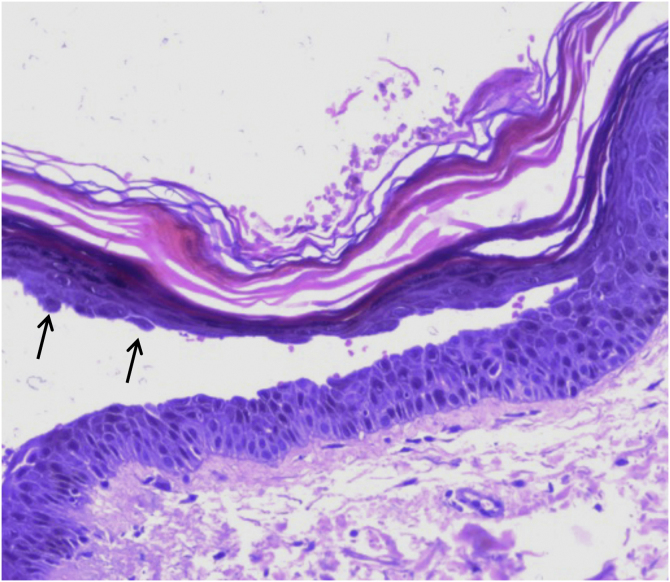
Histopathology showing intraepidermal cleavage and acantholytic keratinocytes (arrows); Hematoxylin & eosin, ×200

Therapy with 60 mg of oral prednisone was implemented but there was acral expansion of the condition in the following six months, leading to lamellar desquamation of the plantar regions (Fig. 1B). Oral methotrexate (15 mg per week) was added to the therapy, which the patient did not tolerate. The condition continued to expand until it became erythrodermic after six months (Fig. 3A). Therapy was then instituted with intravenous (IV) immunoglobulin, at a total dose of 2 g/Kg/cycle, infused on five consecutive days; a total of four cycles were applied with four-week intervals between one and the next. Relevant clinical improvement was observed (Fig. 3B), with no side effects, which allowed a significant reduction in the oral corticosteroid use, which is currently at 5 mg daily, after a favorable 14-month follow-up.

**Figure 3:**
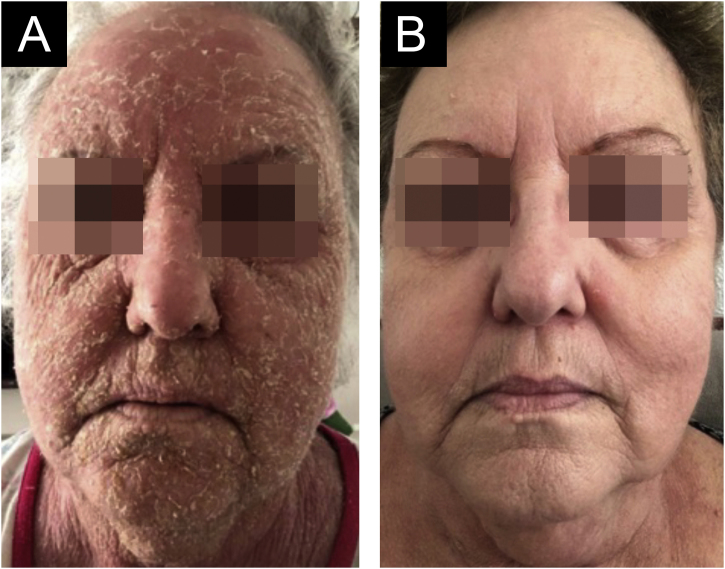
(A) Desquamation of the entire face before treatment. (B) Complete resolution with therapy

The use of IV immunoglobulin is well established in autoimmune diseases, including pemphigus,^3^, ^4^ and is recommended for refractory cases such as this one.^4^ Its action mechanism is probably multiple, with the most relevant being perhaps receptor saturation, with consequent immune cell inhibition. In view of the COVID-19 pandemic, the use of rituximab as a therapeutic alternative has been questioned, due to the intense inhibition of the humoral immune response, and so IV immunoglobulin is an excellent option.

There is only one Brazilian report of an adolescent patient with endemic pemphigus foliaceus,^5^ who also developed the erythrodermic form and was treated with IV immunoglobulin. The case reported herein documents the successful use of this therapy in severe and extensive cases of pemphigus foliaceus, which is rare in this age group.

## Financial support

None declared.

## Authors' contributions

Hiram Almeida Jr: Approval of the final version of the manuscript; design and planning of the study; drafting and editing of the manuscript; collection, analysis, and interpretation of data; effective participation in research orientation; critical review of the manuscript.

Junior Wieczorek: Approval of the final version of the manuscript; design and planning of the study; drafting and editing of the manuscript; collection, analysis, and interpretation of data; critical review of the manuscript.

Mahony Santana: Approval of the final version of the manuscript; design and planning of the study; drafting and editing of the manuscript; collection, analysis, and interpretation of data; critical review of the manuscript.

Celina Leite: Approval of the final version of the manuscript; design and planning of the study; drafting and editing of the manuscript; collection, analysis, and interpretation of data; critical review of the manuscript;

## Conflicts of interest

None declared.
